# *acdh-1* transcripts are expressed in the intestine

**DOI:** 10.17912/W2V365

**Published:** 2017-07-20

**Authors:** Dustin Updike

**Affiliations:** 1 Mount Desert Island Biological Laboratory; Salisbury Cove ME, United States of America

**Figure 1.**
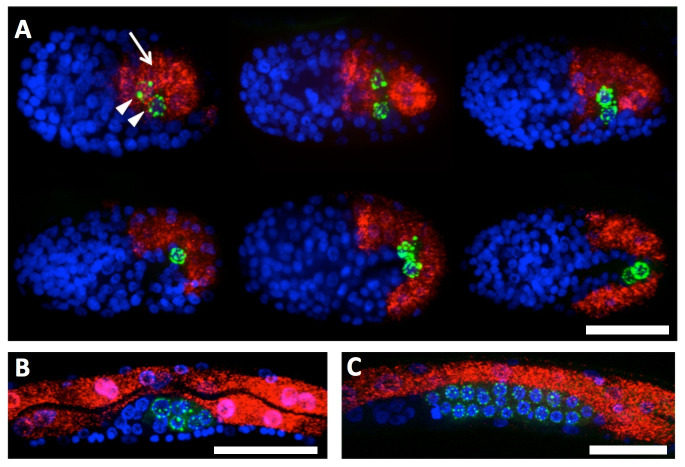


## Description

**​**Stellaris smFISH probes targeting *acdh-1*, a short-chain acyl-CoA dehydrogenase, are shown in red (Cal Fluor 610). DAPI/blue marks embryonic nuclei, and PGL-1::GFP shows the corresponding location of P granules surrounding germ cell nuclei (arrowheads, green). During embryogenesis (A), *acdh-1* expression begins in the E cells (arrow, red). Expression continues in the developing intestine, shown in red, throughout embryogenesis. Intestinal expression of *acdh-1*persists through larval development in the L1 (B) and L2 (C) stages. These results extend previous findings from Arda et al., where *acdh-1 *was shown to be expressed in the adult intestine. scale = 20µ.

## Reagents

The Stellaris *acdh-1* smFISH probe contains 44 oligos labeled with Cal Fluor 610 (sequences available upon request). Staining was performed as described by Ji and van Oudenaarden (2012). In short, PGL-1::GFP embryos and worms were fixed with formaldehyde and ethanol. Hybridization was done in the dark at 37°C for four hours. Cal Fluor 610 coupled probes were designed with the Stellaris Probe Designer from Biosearch Technologies.
